# Biotin-Induced Proximal Renal Tubular Acidosis in an Adolescent Female: Report of a Rare Case With Rechallenge Confirmation

**DOI:** 10.7759/cureus.103396

**Published:** 2026-02-10

**Authors:** Qazi Tauseef Ahmad, Abdul Haseeb, Wajib Ullah, Muzzamil Samad, Ammara Afridi

**Affiliations:** 1 Cardiology, Lady Reading Hospital Medical Teaching Institute, Peshawar, PAK; 2 Internal Medicine, Lady Reading Hospital Medical Teaching Institute, Peshawar, PAK; 3 Internal Medicine, Khyber Medical College, Peshawar, PAK

**Keywords:** arterial blood gases (abgs), biotin supplementation, hypotension, proximal renal tubular acidosis (rta), renal glucosuria, renal tubular acidosis type 2

## Abstract

Proximal renal tubular acidosis (RTA) results from impaired bicarbonate reabsorption in the proximal renal tubule, leading to a normal anion gap metabolic acidosis and electrolyte abnormalities. We report the case of a 16-year-old previously healthy female who presented with recurrent episodes of hypotension, dizziness, and generalized weakness over one month. Laboratory evaluation demonstrated severe hypokalemia, hyperchloremic metabolic acidosis with a normal anion gap, and glucosuria in the absence of hyperglycemia, with preserved renal function. Extensive evaluation for endocrine and autoimmune causes was unrevealing. A detailed review of medications and supplements identified recent initiation of high-dose biotin supplementation (2,500 µg daily) for cosmetic purposes. A strong temporal association was noted between biotin exposure and symptom onset, with rapid clinical and biochemical improvement following discontinuation. Two months later, inadvertent re-exposure to biotin resulted in recurrence of symptoms and laboratory abnormalities, which again resolved promptly after cessation of the supplement. The patient was managed with intravenous fluids, potassium supplementation, and avoidance of biotin, leading to sustained resolution of symptoms. At one-month follow-up, she remained asymptomatic with normal electrolyte levels and preserved renal function. This case identifies biotin as a rare, reversible cause of proximal RTA and underscores the importance of obtaining a thorough supplement history when evaluating patients with otherwise unexplained metabolic acidosis.

## Introduction

Renal tubular acidosis (RTA) encompasses a group of disorders characterized by impaired renal acid handling, resulting in metabolic acidosis with a normal anion gap despite preserved glomerular filtration [1]. Proximal (type 2) RTA results from defective bicarbonate reabsorption in the proximal tubule, often accompanied by other features of proximal tubular dysfunction, including glucosuria, phosphaturia, and aminoaciduria, collectively termed Fanconi syndrome when complete [2].

Acquired causes of proximal RTA include medications (carbonic anhydrase inhibitors, Ifosfamide, tenofovir), heavy metal toxicity, paraproteinemia, and systemic disorders [3]. Drug-induced RTA typically resolves following withdrawal of the offending agent, making prompt recognition clinically important [4].

Biotin (vitamin B7, water-soluble) is widely consumed in supraphysiological doses (often 2500-10,000 µg daily, versus the recommended daily allowance of 30 µg) for purported benefits to hair, skin, and nail health [5]. While biotin is well-recognized for causing interference with immunoassays, direct renal tubular toxicity has rarely been reported in the medical literature [6].

We describe a case of reversible proximal RTA associated with biotin supplementation, confirmed through inadvertent rechallenge, and discuss the clinical implications, given the widespread over-the-counter availability of this supplement.

## Case presentation

A 16-year-old female with no significant past medical history presented with a 1-month history of episodic dizziness, generalized weakness, and documented hypotension (home BP readings 80-90/40-50 mmHg). Episodes initially responded to intravenous fluids but became progressively more frequent, prompting hospital admission. She denied fever, weight loss, polyuria, polydipsia, vomiting, diarrhea, or prescription medication use. No chronic illness, infections, or family history of renal or endocrine disorders were reported. A detailed medication and supplement history revealed that she had initiated oral biotin supplementation at a dose of 2,500 µg daily, approximately four weeks before symptom onset. This dose, while supraphysiological relative to the recommended daily allowance, falls within the range of commercially available over-the-counter hair and nail supplements, which the patient had purchased for cosmetic purposes. Biotin exposure was initially not disclosed until directed questioning by the medical team.

On examination at the time of admission, the patient was alert but mildly fatigued. Blood pressure was 80/60 mmHg with a pulse rate of 88/min. Systemic examination was otherwise unremarkable. There was no skin hyperpigmentation, buccal pigmentation, or other stigmata of adrenal insufficiency. Body weight was appropriate for age.

Initial laboratory evaluation demonstrated severe hypokalemia (1.81 mmol/L), hyperchloremia, and a normal anion gap metabolic acidosis, consistent with a primary metabolic process with appropriate respiratory compensation. Renal function remained preserved throughout admission, with stable serum creatinine and blood urea levels. The trends in electrolytes, arterial blood gas values, and biochemical parameters are summarized in Table 1.

**Table 1:** Serum Biochemistry and Arterial Blood Gas Analysis *Serum potassium after intravenous potassium replacement pH, potential of hydrogen; PaCO₂, partial pressure of arterial carbon dioxide; HCO₃⁻, bicarbonate; ALT, alanine aminotransferase; GPT, glutamate pyruvate transaminase.

Parameter	Reference Range	Day 1 (Admission)	Day 3	Day 6	2-Week Follow-Up
Sodium (mmol/L)	135-150	146.6	138	140	139
Potassium (mmol/L)	3.5-5.1	1.81 / 2.69*	3.64	2.84	3.7
Chloride (mmol/L)	98-107	115	109	105	101
pH	7.35-7.45	7.40	7.45	7.46	7.41
PaCO₂ (mmHg)	35-45	24.8	25.7	43.4	39
HCO₃⁻ (mmol/L)	21-28	14.9	20.6	28.0	24.3
Base excess (mmol/L)	−2 to +3	−8.6	−5.2	+5.7	+2
Serum creatinine (mg/dL)	0.6-1.2	0.6	0.58	0.42	0.51
Blood urea (mg/dL)	15-40	15.39	15.24	28.36	26.82
Total bilirubin(mg/dL)	0.1-1.0	0.6	0.2	-	-
ALT/GPT (U/L)	10-50	18	16	-	-
Alkaline Phosphatase (U/L)	<187	53	54	-	-

Urinalysis revealed marked glucosuria in the absence of hyperglycemia, with a normal HbA1c (5.6%), supporting proximal tubular dysfunction. Urine pH was acidic (pH 5.0), indicating preserved distal urinary acidification and arguing against distal (type 1) renal tubular acidosis. There was no proteinuria, hematuria, or evidence of urinary tract infection. These urinalysis findings are summarized in Table 2.

**Table 2:** Urinalysis RBCs, red blood cells; WBCs, white blood cells; HPF, high-power field; pH, potential of hydrogen.

Parameter	Reference Range	Day 1 (Admission)	Day 6	2-week follow-up
Specific gravity	1.005-1.030	1.025	1.021	1.015
Glucose	Negative	++++	Trace	Negative
Proteins	Negative	Negative	Negative	Negative
pH	4.5-8.0	5	6	7
RBCs (/HPF)	0-2	Nil	2-3	Nil
WBCs (/HPF)	0-5	3	4-5	0
Nitrites	Negative	Negative	Negative	Negative
Leukocyte esterase	Negative	Negative	Negative	Negative

Twenty-four-hour urinary electrolyte analysis demonstrated increased urinary sodium excretion with normal urinary calcium and potassium levels, a pattern compatible with proximal tubular solute wasting. In conjunction with glucosuria and metabolic acidosis, these findings suggested incomplete Fanconi-type proximal tubular dysfunction. The detailed 24-hour urinary electrolyte results are presented in Table 3.

**Table 3:** 24-Hour Urinary Electrolytes

Parameter	Reference Range	Results
Urinary Sodium (mmol/24hr)	40-220	180
Urinary Calcium (mmol/24hr)	100-300	236
Urinary Potassium (mmol/24hr)	25-125	50.6
Urinary Chloride (mmol/24hr)	110-250	209

Endocrine evaluation, including serum cortisol, thyroid-stimulating hormone, glycated hemoglobin (HbA1c), calcium, parathyroid hormone, and vitamin D levels, was within reference ranges, effectively excluding adrenal insufficiency and other endocrine causes. Renal ultrasonography demonstrated structurally normal kidneys with preserved cortical thickness and no evidence of obstruction. The detailed endocrine laboratory results are summarized in Table 4.

**Table 4:** Endocrinology Workup TSH, thyroid-stimulating hormone; PTH, parathyroid hormone; HbA1c, glycated hemoglobin.

Parameter	Reference Range	Results
8 am Cortisol (mcg/dl)	5.27-22.45	10.07
25-Hydroxy Vitamin D(ng/mL)	40-100	29.37
TSH (μU/mL)	0.4-4.0	2.5
HbA1C (%)	<5.7	5.6
Serum Calcium (mg/dl)	8-10	8.6
PTH Intact (pg/ml)	10-60	16.47

The complete blood count (CBC) remained essentially within normal limits throughout the clinical course. Platelet counts were mildly reduced during hospitalization but normalized on follow-up. Overall, the CBC findings were unremarkable and did not suggest an underlying hematologic abnormality, as shown in Table 5.

**Table 5:** Complete Blood Count WBC, white blood cell count; MCV, mean corpuscular volume

Parameter	Reference Range	Day 1 (Admission)	Day 3	2-Week Follow-Up
WBC (×10³/µL)	4-11	6.75	11.8	5.8
Hemoglobin (g/dL)	11.5-17.5	12.1	11.3	12.6
MCV (fL)	76-96	91.3	91.9	92.6
Platelets (×10³/µL)	150-450	120	109	166

Twenty-four-hour ambulatory blood pressure monitoring (Holter) documented frequent hypotensive episodes on the first day of admission, consistent with the patient’s reported symptomatic hypotension; no orthostatic hypotension was observed.

Treatment and clinical management

The patient received careful blood pressure monitoring, intravenous fluid resuscitation with normal saline, and aggressive potassium replacement for severe hypokalemia. Empiric hydrocortisone was administered while adrenal insufficiency was investigated, and later discontinued following normal serum cortisol levels. Serial monitoring of electrolytes and arterial blood gases guided ongoing therapy.

Once biotin exposure was identified, management transitioned to permanent biotin cessation, oral rehydration, and oral potassium supplementation, while hydrocortisone was discontinued. Clinical and biochemical improvement was rapid following cessation of biotin supplementation and supportive management. Within 48-72 hours, hypotensive episodes resolved, serum potassium normalized, and bicarbonate levels improved. Glucosuria progressively resolved, becoming trace by day 6 and completely absent at the 2-week follow-up. The patient reported complete resolution of dizziness and fatigue and returned to normal daily activities without limitation.

Approximately two months later, inadvertent re-exposure to biotin resulted in recurrence of identical symptoms and biochemical abnormalities, which again resolved promptly after permanent discontinuation of the supplement.

At one month following final cessation, the patient remained asymptomatic with normal serum electrolytes, acid-base status, and urinalysis. Ongoing follow-up was arranged with periodic monitoring of serum electrolytes and renal function, in accordance with standard practice for recovered proximal renal tubular dysfunction. The patient did not experience any long-term complications. She is alive and well, and the outcome was not associated with mortality.

The clinical course, including biotin exposure, symptom onset, hospitalization, recovery, and recurrence after inadvertent re-exposure, is summarized in the timeline shown in Table 6.

**Table 6:** Clinical Timeline Illustrating Biotin Exposure, Symptom Onset, Recovery Following Discontinuation, and Recurrence Upon Inadvertent Re-Exposure.

Time Point	Clinical Events
Day 0	Biotin supplementation initiated - 2,500 µg/day
Week 4	Symptom onset - Dizziness, hypotension, generalized weakness
Week 5	Hospital admission - Biotin discontinued
Within 48–72 hours of admission	Rapid symptom improvement
2 weeks post-discharge	Complete clinical and biochemical resolution
2 months post-discharge	Inadvertent biotin re-exposure - Recurrence of symptoms; biotin discontinued again during outpatient visit
1-month follow-up after re-exposure	Clinical recovery - Patient asymptomatic; Normal electrolytes and renal function

## Discussion

The initial presentation of recurrent hypotension, severe hypokalemia, and normal anion gap metabolic acidosis prompted consideration of several differential diagnoses, including renal tubular acidosis, adrenal insufficiency, diabetes mellitus, and drug- or toxin-induced tubular injury. Distal (type 1) renal tubular acidosis was considered but deemed unlikely because of appropriately acidic urine (pH 5.0), indicating preserved distal urinary acidification [7]. In contrast, the combination of metabolic acidosis, severe hypokalemia, glucosuria in the absence of hyperglycemia, and preserved renal function strongly suggested proximal tubular dysfunction. Adrenal insufficiency was an important early consideration given the hypotension; however, normal serum cortisol levels effectively excluded this diagnosis. Furthermore, the presence of hypokalemia rather than the more typical hyperkalemia seen in adrenal insufficiency further argued against this diagnosis in our patient, as aldosterone deficiency in adrenal insufficiency is classically associated with impaired potassium excretion leading to hyperkalemia [8]. Similarly, diabetes mellitus was ruled out by normal blood glucose levels and HbA1c, confirming that the glucosuria was renal rather than glycemic in origin.

With autoimmune, endocrine, and structural renal causes excluded, attention shifted toward an acquired and potentially reversible cause of proximal tubular injury. A detailed medication and supplement history revealed recent initiation of high-dose biotin supplementation, establishing a temporal relationship between exposure and symptom onset. The rapid resolution of clinical and biochemical abnormalities following biotin withdrawal, together with recurrence upon inadvertent rechallenge, confirmed biotin-induced proximal renal tubular acidosis as the final diagnosis.

This case fulfills established criteria for a drug-induced adverse reaction through multiple lines of evidence. The temporal relationship was clear, as the patient developed symptoms approximately four weeks after initiating high-dose biotin (2500 µg/day), a dose commonly marketed for cosmetic purposes but 83-fold higher than the recommended daily allowance [9]. Biochemically, the patient demonstrated classic features of proximal (type 2) renal tubular acidosis, including non-anion gap metabolic acidosis, hypokalemia due to urinary potassium wasting, inappropriate glucosuria with normoglycemia, preserved glomerular filtration, and relatively acidic urine pH, reflecting intact distal acidification capacity [2]. Complete clinical and biochemical normalization occurred within one week of biotin cessation, including correction of hypokalemia, resolution of metabolic acidosis, and disappearance of glucosuria. The causal relationship was further strengthened by inadvertent reintroduction of biotin, which led to recurrence of identical clinical symptoms and laboratory abnormalities. According to the Naranjo Adverse Drug Reaction Probability Scale, this case would be classified as “definite” (score ≥9) given the positive rechallenge [10].

While the precise mechanism of biotin-induced proximal tubular dysfunction remains speculative, several hypotheses warrant consideration. Biotin may interfere with proximal tubular transporters, such as the sodium-glucose cotransporter 2 (SGLT2) or the sodium-bicarbonate cotransporter (NBC1), mimicking the effect of SGLT2 inhibitors [11]. Alternatively, supraphysiological biotin exposure may contribute to proximal tubular dysfunction by affecting cellular metabolic processes, potentially impairing adenosine triphosphate (ATP)-dependent transport mechanisms within proximal tubular epithelial cells [4]. To our knowledge, this represents the first well-documented case of biotin-associated proximal renal tubular acidosis with positive rechallenge confirmation. Although biotin's interference with laboratory assays, particularly thyroid function, troponin, and vitamin D assays, is well-established [6], reports of direct renal tubular effects are exceedingly rare. The widespread availability of high-dose biotin supplements without prescription, combined with aggressive marketing for cosmetic indications, raises a significant public health concern, as many consumers and healthcare providers may be unaware of potential metabolic complications beyond assay interference.

This case report has several inherent limitations. As a single-patient observation, a definitive dose-response relationship between biotin exposure and proximal tubular dysfunction cannot be established, and individual susceptibility factors cannot be assessed. The exact mechanism of injury remains speculative, as no histopathological or mechanistic studies were performed. Additionally, complete characterization of proximal renal tubular dysfunction was limited by the unavailability of fractional bicarbonate excretion and aminoaciduria testing. A further methodological limitation is the potential for biotin interference with streptavidin-biotin-based immunoassays; high-dose biotin is known to interfere with competitive immunoassays, leading to falsely elevated results for analytes such as serum cortisol, which may complicate biochemical evaluation for adrenal insufficiency [6]. Accordingly, the cortisol value should be interpreted with caution. Nevertheless, clinically significant adrenal insufficiency was considered highly unlikely based on the absence of supportive clinical features, the presence of hypokalemia rather than hyperkalemia, and the rapid, sustained resolution of hypotension and metabolic abnormalities following correction of electrolyte disturbances and permanent cessation of biotin without the need for ongoing glucocorticoid therapy.

## Conclusions

This case highlights a rare but clinically significant association between high-dose biotin supplementation and reversible proximal renal tubular acidosis, expanding the spectrum of recognized adverse effects of a commonly perceived benign supplement. It reinforces the critical importance of comprehensive medication reconciliation that explicitly includes over-the-counter and “natural” products, particularly in younger patients presenting with unexplained non-anion gap metabolic acidosis. The clear temporal relationship, biochemical resolution following cessation, and inadvertent rechallenge provide compelling evidence of causality and emphasize the value of diagnostic stewardship in preventing unnecessary invasive investigations and prolonged immunosuppressive therapy. As the use of supraphysiological vitamin supplementation continues to increase, heightened clinician awareness and targeted patient education are essential to mitigate avoidable iatrogenic harm and ensure timely, cost-effective care.
